# Heterogeneity in plasma p‐tau217 response and its association with cognitive trajectories under lecanemab treatment

**DOI:** 10.1002/alz.71705

**Published:** 2026-07-31

**Authors:** Sung Hoon Kang, Yu Jeong Park, Seungyun Lee, Jimin Kang, Seongin Lee, Eun Seong Lee, Hye Na Jung, Inseon Ryoo, Hyundoo Hwang, Kwangho Choi, Jae Seon Eo, Sang‐Il Suh, Kyungmi Oh, Seong‐Beom Koh

**Affiliations:** ^1^ Department of Neurology Korea University Guro Hospital, Korea University College of Medicine Seoul South Korea; ^2^ Department of Medicine Korea University College of Medicine Seoul South Korea; ^3^ Department of Nuclear Medicine Korea University Guro Hospital Korea University College of Medicine Seoul South Korea; ^4^ Department of Radiology Korea University Guro Hospital, Korea University College of Medicine Seoul South Korea; ^5^ Bredis Healthcare Inc. Seoul South Korea

**Keywords:** Alzheimer's disease, biomarker trajectories, lecanemab, plasma p‐tau217, treatment response

## Abstract

**INTRODUCTION:**

Plasma phosphorylated tau 217 (p‐tau217) is a promising biomarker for monitoring treatment response in Alzheimer's disease (AD), but its longitudinal dynamics and clinical relevance remain unclear.

**METHODS:**

In this prospective real‐world study, 153 patients with early AD receiving lecanemab were analyzed. Longitudinal changes in plasma p‐tau217 were assessed, and trajectory patterns were identified using clustering and slope‐based approaches. Associations with baseline factors and cognitive outcomes were evaluated.

**RESULTS:**

Plasma p‐tau217 levels decreased significantly from 3 months, with the greatest decline between 3 and 6 months, followed by a plateau. Two distinct trajectory groups were identified. Patients in the greater reduction group showed more favorable cognitive trajectories, particularly slower progression in Clinical Dementia Rating–Sum of Boxes (CDR‐SB) scores. Hypertension was associated with a diminished biomarker response.

**DISCUSSION:**

These findings support plasma p‐tau217 as an early pharmacodynamic biomarker and highlight its potential role in guiding individualized treatment strategies in routine clinical practice.

## Highlights

- Plasma phosphorylated tau 217 (p‐tau217) levels showed an early and significant reduction following lecanemab treatment, with the greatest decline between 3 and 6 months
- Distinct trajectory patterns of p‐tau217 change highlight heterogeneity in biomarker response
- Greater reduction in p‐tau217 was associated with more favorable cognitive trajectories, particularly slower progression in Clinical Dementia Rating–Sum of Boxes (CDR‐SB)
- Baseline hypertension was associated with a diminished biomarker response, suggesting that subtle vascular dysfunction, including impaired glymphatic clearance, may modulate treatment effectiveness
- Plasma p‐tau217 may serve as an early pharmacodynamic biomarker to guide individualized treatment strategies

## 1 BACKGROUND

Alzheimer's disease (AD) is a progressive neurodegenerative disorder characterized by the accumulation of amyloid beta (Aβ) and tau pathology, ultimately leading to cognitive decline.^1^ The recent introduction of anti‐amyloid therapies, particularly lecanemab, has shifted the treatment paradigm from symptomatic management toward disease modification in early AD.^2^, ^3^ However, despite these advances, predicting individual treatment response remains challenging in real‐world clinical practice, underscoring the need for reliable biomarkers that can monitor therapeutic effects and guide clinical decision‐making.

Among emerging blood‐based biomarkers, plasma phosphorylated tau 217 (p‐tau217) has shown high diagnostic accuracy for AD and is considered a T1 biomarker reflecting both amyloid and tau pathology.^4^, ^5^ In the early stages of the disease, p‐tau217 levels are more closely associated with amyloid burden while also capturing downstream tau‐related changes.^5^ Recent evidence suggests that p‐tau217 may serve as a pharmacodynamic marker of anti‐amyloid treatment response.^3^, ^6^ In particular, a recent real‐world study has demonstrated that plasma p‐tau217 levels decrease over time following therapy and are associated with cognitive outcomes, supporting its potential role as a dynamic biomarker of disease modification.^7^ Despite these advances, important questions remain. Prior study has primarily focused on average longitudinal changes in p‐tau217, typically assessed as absolute changes from baseline, with limited exploration of inter‐individual variability or slope‐based trajectory patterns. Of note, baseline clinical and biological factors associated with the magnitude and pattern of p‐tau217 reduction have not been well characterized. Finally, the clinical relevance of different p‐tau217 trajectory patterns, particularly in relation to cognitive outcomes, remain to be further elucidated.

In this prospective real‐world study, we aimed to characterize longitudinal changes in plasma p‐tau217 levels in patients with early AD treated with lecanemab, identify distinct trajectory patterns of biomarker response, and evaluate their associations with baseline clinical factors and cognitive outcomes. We sought to provide insights into treatment response heterogeneity and the potential role of plasma p‐tau217 as a biomarker for guiding individualized therapeutic strategies.

## 2 METHODS

### 2.1 Study design and participants

This study was conducted as part of the K‐LEARN (Korean Lecanemab Real‐world Evidence and Response Network) cohort, a prospective real‐world registry of patients receiving lecanemab at a tertiary referral memory clinic at Korea University Guro Hospital, Seoul, Korea. At baseline, we prospectively recruited 153 patients across the AD continuum, including patients with early AD (mild cognitive impairment [MCI] due to AD and mild AD dementia) and individuals with subjective cognitive decline (SCD), who initiated lecanemab treatment at this clinic between December 2024 and March 2026. All patients fulfilled the following criteria for a diagnosis of early AD: (1) subjective cognitive complaints from the patients or caregivers; (2) objective cognitive decline in at least one domain of memory, language, visuospatial, or frontal function evaluated by comprehensive neuropsychological battery; (3) a severity of cognitive impairment consistent with MCI or mild dementia, defined by a global Clinical Dementia Rating (CDR) score of 0.5 or 1; and (4) evidence of amyloid pathology confirmed by amyloid positron emission tomography (PET). All individuals with SCD met the following criteria: persistent subjective cognitive complaints in the absence of objective cognitive impairment on comprehensive neuropsychological testing, a global CDR score of 0.5, and evidence of cerebral amyloid pathology confirmed by amyloid PET. Although SCD is not a standard indication in all Appropriate Use Recommendation (AUR)^8^, ^9^ frameworks, selected SCD patients with confirmed amyloid pathology were included based on clinical judgment in a real‐world setting.

At baseline, all participants underwent comprehensive clinical and laboratory evaluations, including neurological examination, routine laboratory tests, standardized neuropsychological assessment using the Seoul Neuropsychological Screening Battery,^10^, ^11^ Korean version of the Mini‐Mental State Examination, Second Edition (MMSE),^12^ brain magnetic resonance imaging (MRI), amyloid PET, and apolipoprotein E (*APOE*) genotyping. Blood samples were also collected for analysis of AD‐related biomarkers, including plasma p‐tau‐217.^13^

Patients were excluded if they had conditions considered inappropriate for lecanemab treatment, including recent stroke, transient ischemic attack, seizure, or bleeding disorders within the past 12 months; active anticoagulant or thrombolytic therapy; contraindications to MRI; significant hemorrhagic lesions on MRI; or unstable psychiatric illness.

The study protocol was approved by the institutional review board of Korea University Guro Hospital, and written informed consent was obtained from all participants.

**RESEARCH IN CONTEXT**

1. **Systematic review**: Anti‐amyloid therapies, including lecanemab, have demonstrated clinical efficacy in randomized trials such as the Clarity AD phase 3 clinical trial (CLARITY AD). Plasma phosphorylated tau (p‐tau217) has emerged as a promising blood‐based biomarker reflecting both amyloid and tau pathology and has been proposed as a pharmacodynamic marker of treatment response. However, real‐world evidence on longitudinal p‐tau217 dynamics and their clinical relevance remains limited.
2. **Interpretation**: In this prospective real‐world study, plasma p‐tau217 levels showed an early and significant reduction following lecanemab treatment, with distinct trajectory patterns observed. Greater reduction in p‐tau217 was associated with more favorable cognitive trajectories, particularly slower progression in Clinical Dementia Rating–Sum of Boxes (CDR‐SB) scores. Baseline hypertension was associated with a diminished biomarker response, suggesting a potential influence of vascular burden on treatment effectiveness.
3. **Future directions**: Larger, multicenter studies with longer follow‐up are needed to validate biomarker‐based response patterns and establish clinically meaningful thresholds. Integration of multimodal biomarkers, including tau and cerebrovascular measures, will be essential to better characterize treatment heterogeneity and advance biomarker‐guided precision medicine in Alzheimer's disease.

### 2.2 Lecanemab treatment protocol

Lecanemab was administered intravenously every 2 weeks at a dose of 10 mg/kg, diluted in 0.9% sodium chloride, in accordance with regulatory guidance^14^ and clinical practice recommendations.^8^ Each infusion lasted ≈1 h and was followed by a monitoring period of 6 h after the first infusion, 2 h after the second and third infusions, and 1 h for subsequent infusions.

### 2.3 MRI acquisition

Baseline MRI was performed within 2 months prior to treatment initiation. Follow‐up MRI scans were conducted at 2, 3, 6, and 12 months, with additional imaging obtained if clinically indicated, and further follow‐up planned at 18 months. Detailed imaging protocols are provided in the Supplementary Methods.

### 2.4 Amyloid PET acquisition and quantification

Amyloid PET imaging using 18F‐florbetaben was performed at baseline to confirm amyloid positivity. Follow‐up imaging is planned at 18 months to assess longitudinal changes in amyloid burden. PET images were interpreted independently by two readers with consensus adjudication.^15^, ^16^ Quantitative analysis was performed using the Centiloid (CL) values based on standardized procedures. Detailed acquisition and processing methods are described in the Supplementary Methods.

### 2.5 Measurement of cognitive trajectory

Cognitive assessments were conducted at baseline and at 3, 6, and 12 months after treatment initiation, with additional follow‐up planned at 18 months. Evaluations included the MMSE, Clinical Dementia Rating–Sum of Boxes (CDR‐SB), and comprehensive neuropsychological testing. The full neuropsychological battery was administered at baseline and 12 months, whereas MMSE and CDR‐SB were assessed at all follow‐up visits.

### 2.6 Plasma collection and plasma biomarker assays

All participants underwent plasma collection at baseline (the day before lecanemab initiation) and at 3 and 6 months after treatment initiation, with additional assessments planned at 12 and 18 months. At each time point, 20 mL of peripheral blood was collected from each participant and placed into two 1.0 M ethylenediaminetetraacetic acid (EDTA)–containing tubes, followed by gentle mixing for 5 min. Plasma was separated by centrifugation at 1300 × *g* for 10 min and subsequently aliquoted into 20–24 vials, each containing 0.3 mL. All plasma samples were stored at −80°C until analysis. This procedure was conducted in accordance with the manual for human biospecimen collection and registration of the National Biobank of Korea.

Plasma p‐tau217 concentrations were measured at a certified central laboratory (Bredis Healthcare Inc., Seoul, South Korea). To ensure analytical precision, all assays were performed in an ISO 14644‐1 Class 8 cleanroom (temperature 22 ± 2°C; relative humidity 50 ± 10%) using the Simoa HD‐X Analyzer (Quanterix, Billerica, MA, USA). Plasma p‐tau217 levels were quantified using the ALZpath p‐Tau217 Advantage PLUS Reagent Kit (Quanterix). Following a single freeze–thaw cycle, plasma samples were diluted threefold by the instrument, and all analyses were conducted according to the manufacturer's instructions.

### 2.7 *APOE* genotyping

*APOE* genotyping was performed using baseline blood samples. Participants were classified as ε4 non‐carriers and carriers for subsequent analyses.

### 2.8 Statistical analysis

Continuous and categorical variables are presented as mean ± SD and frequency (%), respectively.

To visualize individual longitudinal trajectories of plasma p‐tau217 levels over time, spaghetti plots were generated with overlaid mean trends and 95% confidence intervals (CIs). To further assess longitudinal changes, linear mixed‐effects models were applied with time as a fixed effect and subject as a random effect to account for within‐subject correlations. Time was treated as a categorical variable (baseline, 3, 6, and 12 months), and pairwise comparisons versus baseline were performed to estimate the magnitude and statistical significance of changes at each follow‐up time point.

To identify distinct patterns of longitudinal change in plasma p‐tau217 levels and explore heterogeneity in treatment response, k‐means clustering was performed using baseline‐normalized, log‐transformed values. Briefly, plasma p‐tau217 concentrations were log‐transformed to reduce skewness, and only participants with available measurements at baseline, 3 months, and 6 months were included. For each participant, changes from baseline were calculated as the difference between log‐transformed values at follow‐up visits and baseline (i.e., ln(p‐tau217 at 3 months) − ln(p‐tau217 at baseline) and ln(p‐tau217 at 6 months) − ln(p‐tau217 at baseline)). These values were standardized and used as input variables for k‐means clustering (*k = *2). The optimal number of clusters was determined based on silhouette coefficients, and a two‐cluster solution was selected considering both clustering performance and clinical interpretability. The resulting clusters were classified as “greater reduction” and “lesser reduction” groups according to the magnitude of p‐tau217 reduction at 6 months.

Baseline clinical characteristics between the two groups were compared using appropriate statistical tests according to variable type. In addition, to identify baseline characteristics associated with membership in the greater reduction group, logistic regression analysis was performed using age, sex, body mass index (BMI), hypertension, diabetes, hyperlipidemia, MCI status, *APOE* ε4 carrier, MMSE, CDR‐SB, CL values, and plasma p‐tau217 levels as predictors. Univariable analyzes were first conducted for each variable, followed by multivariable logistic regression including all covariates to identify independent predictors. Multicollinearity was assessed prior to model fitting.

To further quantify early treatment response, the rate of change in plasma p‐tau217 between baseline and 6 months was calculated for each participant as an individual slope. Linear regression models were applied to evaluate the association between baseline clinical variables and the p‐tau217 slope. Baseline variables included age, sex, BMI, hypertension, diabetes, hyperlipidemia, MCI status, *APOE* ε4 carrier, MMSE, CDR‐SB, CL values, and baseline plasma p‐tau217 levels. Univariable linear regression analyzes were first performed to assess the association between each baseline variable and the p‐tau217 slope. Subsequently, all variables were simultaneously entered into a multivariable linear regression model to identify independent factors associated with the p‐tau217 slope.

To evaluate whether these biological changes translated into clinical outcomes, longitudinal changes in MMSE and CDR‐SB scores were assessed. Spaghetti plots with overlaid mean trajectories and 95% CIs were constructed at 3, 6, and 12 months. Linear mixed‐effects models were used to examine whether trajectory group (greater reduction vs lesser reduction) was associated with differences in the rate of change in MMSE and CDR‐SB scores. A time‐by‐trajectory group interaction term was included to assess differential longitudinal trajectories between groups.

To further investigate the impact of early biomarker response on clinical outcomes, participants were additionally classified using a slope‐based grouping approach derived from changes in plasma p‐tau217 levels between baseline and 6 months. The p‐tau217 slope was calculated for each participant, and a predefined cutoff corresponding to the upper 35% of the slope distribution was applied to define the steeper decline group, with the remaining participants categorized as the slower decline group. This cutoff was selected to match the proportion of patients in the greater reduction group identified in the trajectory‐based analysis. Longitudinal changes in cognitive performance were compared between groups using the linear mixed‐effects models. In contrast to the trajectory‐based analysis, which incorporated longitudinal temporal patterns across baseline, 3‐month, and 6‐month measurements, the slope‐based approach primarily reflected the overall magnitude of change between baseline and 6 months and was performed as a complementary validation analysis.

To further evaluate whether longitudinal p‐tau217 trajectory patterns provided prognostic information beyond baseline disease burden, additional sensitivity analyzes were performed using linear mixed‐effects models incorporating interactions between time and baseline MMSE, baseline CDR‐SB, baseline CL values, and baseline plasma p‐tau217 levels. Baseline factor–based interaction models were first constructed for MMSE and CDR‐SB trajectories after controlling for age, sex, and *APOE* ε4 carrier status. Subsequently, time‐by‐trajectory group interaction terms were added to these models to assess whether longitudinal p‐tau217 trajectory groups provided incremental prognostic information beyond baseline clinical and biomarker characteristics. Model fit was compared between baseline factor–based models and trajectory‐enhanced models using likelihood ratio tests as well as Akaike information criterion (AIC) values.

All reported p‐values were two‐sided, and statistical significance was defined as *p* < 0.05. Missing data were not imputed and were excluded from analyses as appropriate. All statistical analyzes were performed using SAS version 9.4 (SAS Institute Inc., Cary, NC, USA) and R version 4.4.1 (R Foundation for Statistical Computing, Vienna, Austria).

## 3 RESULTS

### 3.1 Baseline characteristics

Among patients who initiated lecanemab treatment, 153 with available baseline plasma p‐tau217 measurements were included in the present analysis. Of these, 81 patients had plasma samples available at baseline, and 3 and 6 months, and were included in the analysis of longitudinal p‐tau217 trajectory patterns. Baseline characteristics of the overall cohort (*n =* 153) are presented in Table S1. The mean age ± SD was 72.7 ± 8.3 years, and 92 patients (60.1%) were female. The mean years of education were 11.8 ± 4.7. With respect to cognitive impairment severity, 117 patients (76.5%) had a global CDR score of 0.5. The mean CDR‐SB score was 3.02 ± 1.95, and the mean MMSE score was 23.6 ± 3.7. *APOE* ε4 carrier accounted for 55.6% of the cohort (85 patients). Comorbidities including hypertension, diabetes, and hyperlipidemia were present in 34.6%, 18.3%, and 43.8% of patients, respectively. The mean CL value was 70.70 ± 35.31, and the mean plasma p‐tau217 level was 1.19 ± 0.61.

### 3.2 Longitudinal changes in plasma p‐tau217 levels

**FIGURE 1:**
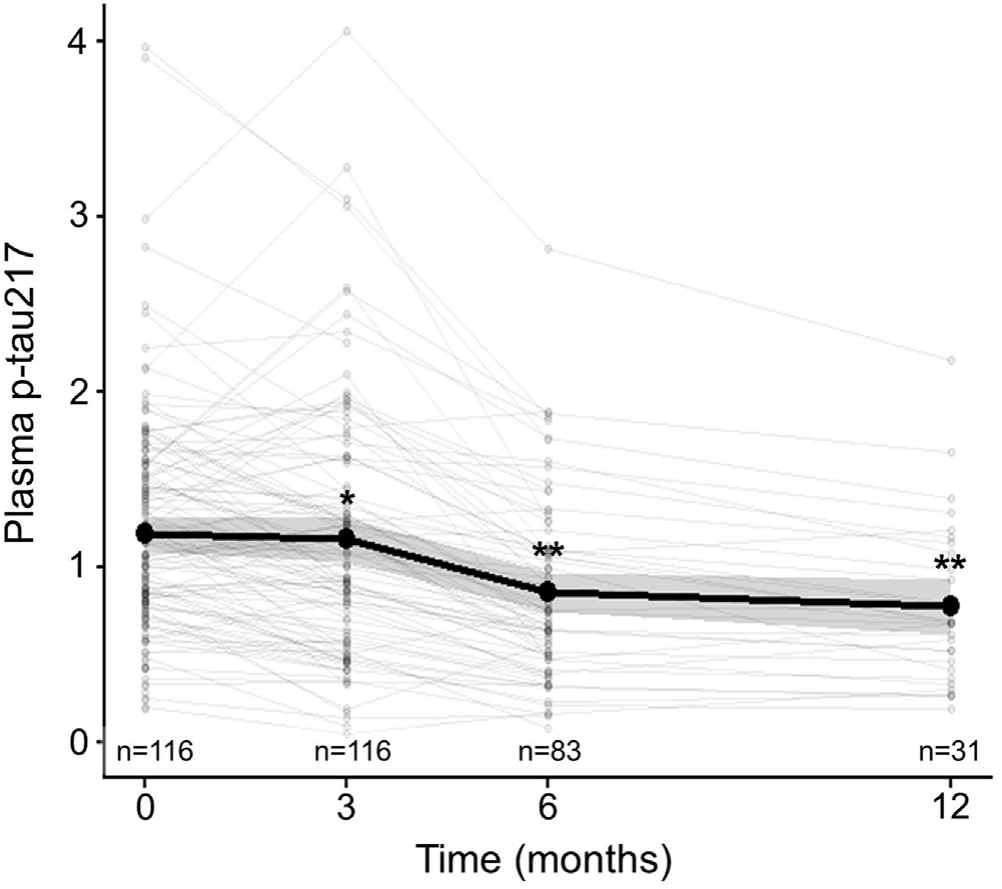
Longitudinal changes in plasma phosphorylated tau 217 (p‐tau217) levels following lecanemab treatment. Spaghetti plots show individual trajectories (gray lines), with mean values and 95% confidence intervals overlaid (black line and shaded area). Plasma p‐tau217 levels decreased significantly over time. Asterisks indicate statistical significance compared with baseline based on linear mixed‐effects models (*p < 0.05; **p < 0.001). Time is shown in months from treatment initiation. Numbers above the x‐axis represent the number of participants with available data at each time point.

Plasma p‐tau217 levels showed a significant decrease over time in linear mixed‐effects models (Figure 1). Post hoc pairwise comparisons demonstrated that p‐tau217 levels were reduced significantly at 3 months compared with baseline (estimate −0.108, *p* = 0.011), with a more pronounced decline observed at 6 months (estimate −0.460, *p <* 0.001). This reduction was sustained and slightly further progressed at 12 months (estimate −0.579, *p <* 0.001). Overall, p‐tau217 levels began to decrease as early as 3 months after treatment initiation, with the greatest magnitude of reduction occurring between 3 and 6 months, followed by a plateauing trend thereafter.

### 3.3 Patterns of longitudinal change in plasma p‐tau217

**FIGURE 2:**
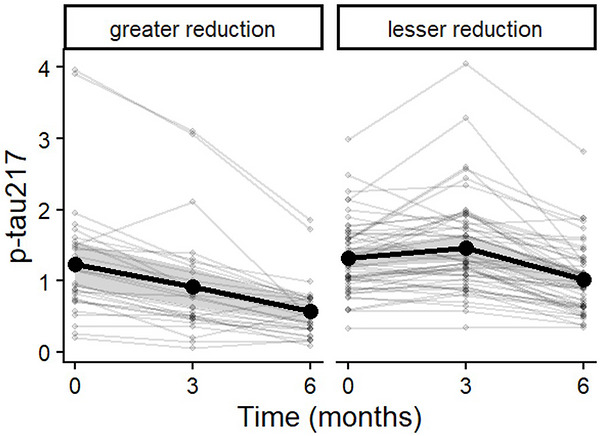
Distinct trajectory patterns of plasma phosphorylated tau 217 (p‐tau217) following lecanemab treatment. Spaghetti plots illustrate individual longitudinal changes in plasma p‐tau217 levels (gray lines), with mean values and 95% confidence intervals overlaid (black lines and shaded areas). Participants were classified into trajectory groups based on longitudinal changes in p‐tau217, defined as the greater reduction group and the lesser reduction group. The greater reduction group showed a marked and consistent decline in p‐tau217 levels over time, whereas the lesser reduction group exhibited relatively attenuated changes with a transient increase at 3 months followed by a modest decline at 6 months. Time is shown in months from treatment initiation.

The k‐means clustering identified two distinct patterns of longitudinal change in plasma p‐tau217 levels. Based on the magnitude of reduction from baseline, patients were categorized into greater reduction and lesser reduction groups. Patients in the greater reduction group demonstrated a consistent and marked decline in plasma p‐tau217 levels from baseline to 6 months, whereas those in the lesser reduction group showed relatively smaller changes, with a transient increase or minimal reduction at 3 months followed by a modest decline at 6 months. These distinct trajectory patterns were clearly visualized in spaghetti plots (Figure 2).

The clustering results indicated a moderate degree of separation between groups, supported by a silhouette score of 0.446. This clustering structure was further supported by principal component analysis, which demonstrated overall separation between the two clusters (Figure S1).

### 3.4 Baseline characteristics according to p‐tau217 trajectory groups

Baseline clinical characteristics were largely comparable between the two groups (Table 1). Patients in the greater reduction group had significantly higher MMSE scores at baseline compared with those in the lesser reduction group (24.7 ± 3.9 vs 22.7 ± 3.2, *p* = 0.016). In contrast, baseline CDR‐SB scores did not differ significantly between the groups (3.05 ± 2.24 vs 3.64 ± 1.87, *p* = 0.231). With respect to biomarker profiles, amyloid burden as measured by CL values was significantly lower in the greater reduction group (64.8 ± 31.0 vs 83.3 ± 34.8, *p* = 0.019), whereas baseline plasma p‐tau217 levels were comparable between groups (*p* = 0.661). The prevalence of hypertension was significantly lower in the greater reduction group compared with the lesser reduction group (10.3% vs 42.3%, *p* = 0.006). Other baseline characteristics, including age, sex, education, BMI, and *APOE* ε4 carrier, did not differ significantly between the groups. Rates of total amyloid‐related imaging abnormalities (ARIA), ARIA with edema/effusion, and ARIA with hemorrhage did not differ significantly between the groups (Table 1).

**TABLE 1:** Baseline clinical and biomarker characteristics according to p‐tau217 trajectory groups.

	Greater reduction (*N =* 29)	Lesser reduction (*N =* 52)	*p*‐value
Age, years	72.5 ± 8.5	71.1 ± 8.7	0.484
Female, *n* (%)	14 (48.3)	35 (67.3)	0.149
Education, years	12.7 ± 4.1	12.2 ± 4.5	0.579
BMI	23.5 ± 3.3	22.9 ± 3.0	0.433
CDR‐SB	3.05 ± 2.24	3.64 ± 1.87	0.221
MMSE	24.7 ± 3.9	22.7 ± 3.2	0.016
*APOE* ε4 carrier	13 (44.8)	29 (55.8)	0.476
Hypertension, *n* (%)	3 (10.3)	22 (42.3)	0.006
Diabetes, *n* (%)	5 (17.2)	6 (11.5)	0.704
Hyperlipidemia, *n* (%)	11 (37.9)	17 (32.7)	0.817
CL values	64.76 ± 31.02	83.32 ± 34.80	0.019
p‐tau217 level	1.23 ± 0.88	1.32 ± 0.52	0.661
Total ARIA	2 (6.9)	8 (15.4)	0.318
ARIA‐E	0 (0.0)	3 (5.8)	0.549
ARIA‐H	2 (6.9)	6 (11.5)	0.705

Values are presented as mean ± SD for continuous variables and number (%) for categorical variables.

The *p*‐values were calculated using the Student's *t*‐test for continuous variables, and the chi‐square test for categorical variables. Fisher's exact test was used for comparisons of ARIA frequencies.

The greater reduction group and lesser reduction group were defined based on longitudinal changes in plasma p‐tau217 levels over the initial 6 months of treatment.

Abbreviations: *APOE* ε4, apolipoprotein E ε4; ARIA, amyloid‐related imaging abnormalities; ARIA‐E, amyloid‐related imaging abnormalities with edema/effusion; ARIA‐H, amyloid‐related imaging abnormalities with hemorrhage; BMI, body mass index; CDR‐SB, Clinical Dementia Rating–Sum of Boxes; CL, Centiloid; MMSE, Korean version of the Mini‐Mental State Examination, Second Edition; p‐tau217, tau phosphorylated at threonine 217.

### 3.5 Baseline predictors of p‐tau217 reduction

**FIGURE 3:**
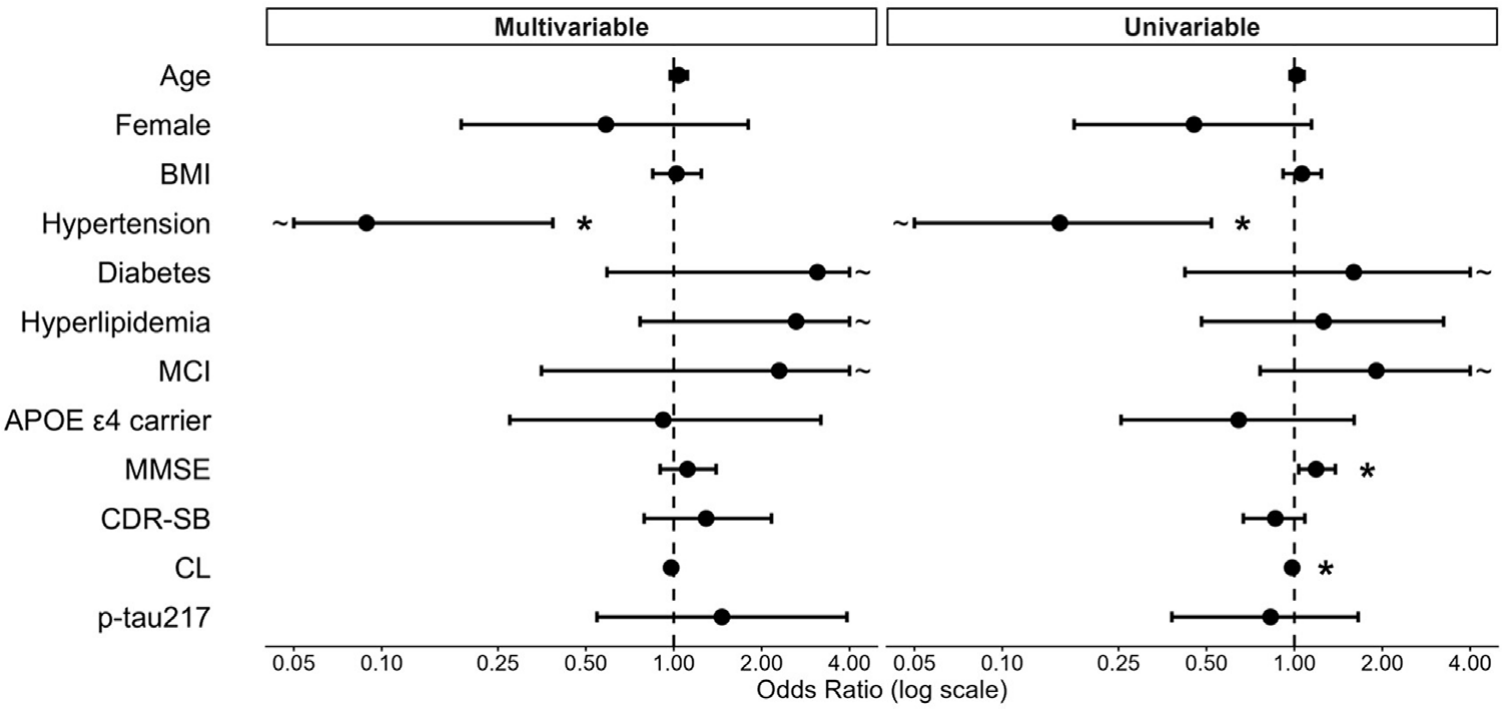
Factors associated with membership in the greater reduction group of plasma p‐tau217 trajectories. Forest plots show the associations between baseline clinical variables and the likelihood of belonging to the greater reduction group (reference: lesser reduction group). Odds ratios (ORs) and 95% confidence intervals are shown for multivariable (left panel) and univariable (right panel) logistic regression analyzes. The dashed vertical line represents the null value (OR = 1). An OR <1 indicates a lower likelihood of belonging to the greater reduction group, whereas an OR >1 indicates a higher likelihood. Hypertension was significantly associated with a reduced likelihood of membership in the greater reduction group. Asterisks (*) denote statistical significance (*p <* 0.05). *APOE* ε4, apolipoprotein E ε4; BMI, body mass index; CDR‐SB, Clinical Dementia Rating–Sum of Boxes; CL, Centiloid; MCI, mild cognitive impairment; MMSE, Korean version of the Mini‐Mental State Examination, Second Edition; OR, odds ratio; p‐tau217, phosphorylated tau 217.

To identify baseline factors associated with p‐tau217 response, we performed complementary analyses using both a trajectory clustering–based approach and a slope‐based approach. Trajectory clustering–based analysis is shown in Figure 3. Univariable logistic regression showed that hypertension was associated with lower odds of belonging to the greater reduction group (odds ratio [OR] 0.16, 95% confidence interval [CI] 0.03–0.52, *p* = 0.006). Higher baseline MMSE scores were associated with increased odds (OR 1.19, 95% CI 1.03–1.38, *p* = 0.019), whereas higher amyloid burden measured by CL values was associated with decreased odds (OR 0.98, 95% CI 0.97–1.00, *p* = 0.024). Other variables, including age, sex, BMI, diabetes, hyperlipidemia, MCI status, *APOE* ε4 carrier, baseline CDR‐SB, and plasma p‐tau217 levels, were not significantly associated with group membership. In multivariable logistic regression analysis, hypertension remained independently associated with a lower likelihood of belonging to the greater reduction group (OR 0.09, 95% CI 0.01–0.39, *p* = 0.003). No other variables, including MMSE and CL values, showed statistically significant associations after adjustment.

In the slope‐based analysis, univariable linear regression demonstrated that older age (*β* = −0.002, *p* = 0.025) and higher baseline MMSE scores (*β* = −0.005, *p* = 0.011) were associated with a steeper decline in plasma p‐tau217 levels. In contrast, hypertension (*β* = 0.033, *p* = 0.022) and higher baseline CDR‐SB scores (*β* = 0.007, *p* = 0.043) were associated with a slower decline in p‐tau217 levels. Other baseline variables were not significantly associated with p‐tau217 slope. In multivariable linear regression analysis, older age (*β* = −0.002, *p* = 0.011) remained independently associated with a steeper decline in plasma p‐tau217 levels, whereas hypertension (*β* = 0.039, *p* = 0.014) remained independently associated with a slower decline in plasma p‐tau217 levels. No other variables were independently associated with the p‐tau217 slope after adjustment (Figure S2).

### 3.6 Association between p‐tau217 trajectories and cognitive outcomes

Longitudinal changes in cognitive performance were assessed using MMSE and CDR‐SB scores over 12 months. MMSE scores remained stable over time, with no significant differences observed at 3, 6, or 12 months compared with baseline (Figure 3). In contrast, CDR‐SB scores showed a gradual increase over time, indicating progressive cognitive decline (Figure 3).

**FIGURE 4:**
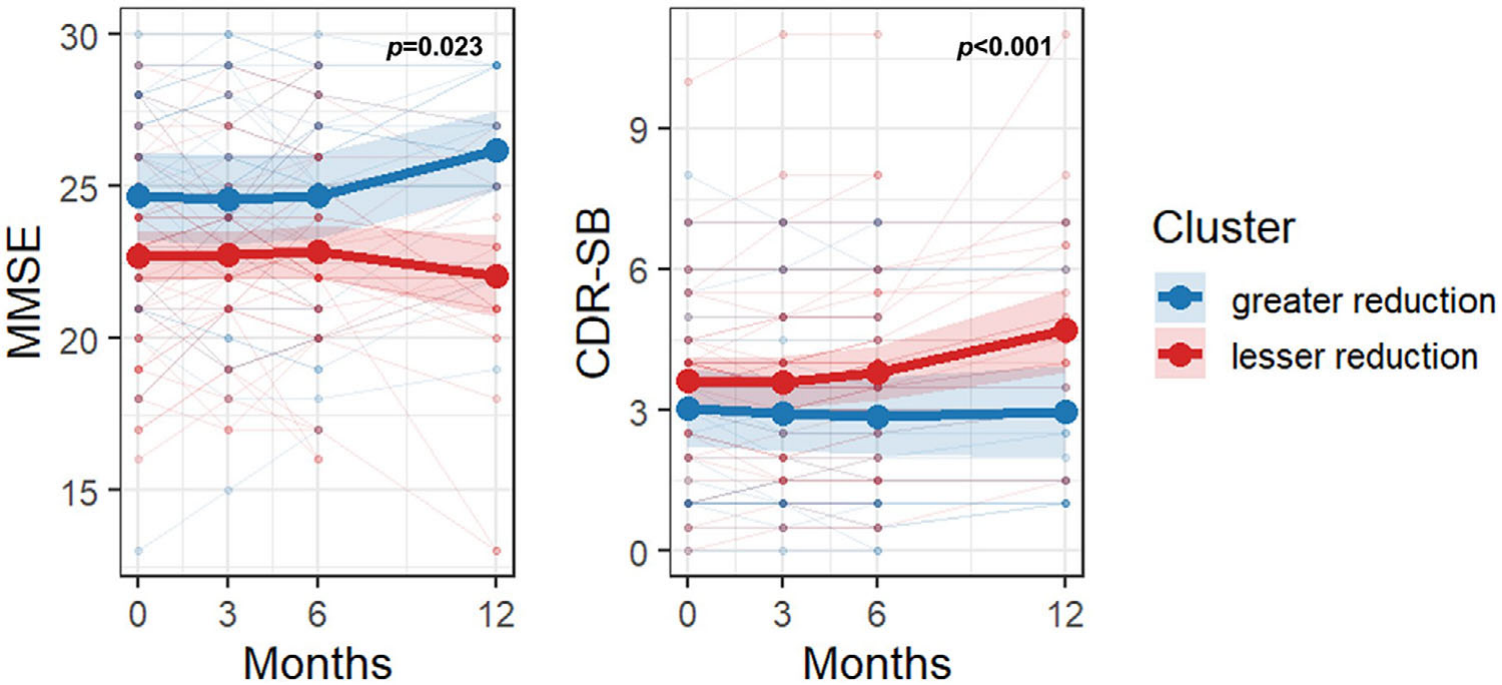
Longitudinal cognitive trajectories according to plasma p‐tau217 trajectory group. Spaghetti plots illustrate individual longitudinal changes in MMSE (left) and CDR‐SB (right) scores (faint lines), with group‐level mean trajectories and 95% confidence intervals overlaid (solid lines and shaded areas). Participants were stratified into p‐tau217 trajectory groups (greater reduction vs lesser reduction). Linear mixed‐effects models adjusted for age, sex, *APOE* ε4 carrier, baseline CDR‐SB scores, baseline CL values, and baseline plasma p‐tau217 levels were used to assess differences in longitudinal cognitive changes between groups. The *p*‐values represent the significance of the interaction between time and trajectory groups, indicating differential rates of change over time between groups. A significant interaction indicates that the rate of cognitive change differed between trajectory groups over time. *APOE* ε4, apolipoprotein E ε4; CDR‐SB, Clinical Dementia Rating–Sum of Boxes; CL, Centiloid; MMSE, Korean version of the Mini‐Mental State Examination, Second Edition; p‐tau217, tau phosphorylated at threonine 217.

Regarding cognitive trajectories stratified by the p‐tau217 trajectory group, patients in the greater reduction group exhibited more favorable cognitive trajectories compared with those in the lesser reduction group (Figure 4). Specifically, MMSE scores remained stable or slightly improved in the greater reduction group, whereas a gradual decline was observed in the lesser reduction group (*p* = 0.023). In parallel, CDR‐SB scores increased more slowly in the greater reduction group, whereas a more pronounced worsening was observed in the lesser reduction group (*p* < 0.001).

As a complementary validation of these findings, we performed additional analyses using a slope‐based grouping approach derived from the 0–6 month change in plasma p‐tau217 levels. Participants were categorized into steeper and slower decline groups based on the magnitude of p‐tau217 slope, using a predefined cutoff corresponding to the upper 35% of the slope distribution to match the proportion of patients classified into the greater reduction group in the trajectory‐based analysis. Consistent with the primary analysis, patients in the steeper decline group showed a trend toward more favorable MMSE trajectories compared with those in the slower decline group, although this did not reach statistical significance (*p* = 0.078). In contrast, CDR‐SB trajectories differed significantly between the groups, with a slower rate of increase observed in the steeper decline group (*p* = 0.006; Figure S3).

### 3.7 Incremental prognostic value of p‐tau217 trajectory groups beyond baseline factors

In baseline factor–based interaction models, higher baseline MMSE scores (*p* < 0.001) and lower baseline CDR‐SB scores (*p* = 0.010) were associated with slower longitudinal decline in MMSE scores (Table S2). For CDR‐SB trajectories, higher baseline MMSE scores (*p* = 0.013) were associated with slower longitudinal progression in CDR‐SB scores (Table S2). Of note, after additionally incorporating p‐tau217 trajectory groups into these baseline factor–based models, the time‐by‐trajectory group interaction remained significantly associated with longitudinal changes in both MMSE (*p* < 0.001) and CDR‐SB scores (*p* = 0.001).

Furthermore, inclusion of the time‐by‐trajectory group interaction significantly improved model fit compared with baseline factor–based interaction models for both MMSE (AIC 1123.8 vs 1113.0; likelihood ratio test *χ^2^ *= 14.80, *p =* 0.001) and CDR‐SB (AIC 548.5 vs 534.8; *χ^2^ *= 17.61, *p <* 0.001). These findings support the incremental prognostic value of longitudinal plasma p‐tau217 response beyond baseline clinical and biomarker characteristics.

## 4 DISCUSSION

In this prospective real‐world study, we characterized early biomarker dynamics and their clinical implications in patients with early AD treated with lecanemab, with a particular focus on plasma p‐tau217 trajectories and their association with treatment response. The major findings were as follows: (1) plasma p‐tau217 levels showed a significant and early reduction beginning at 3 months after treatment initiation, with the greatest decline observed between 3 and 6 months, followed by a plateau thereafter; (2) distinct trajectory patterns of p‐tau217 change were identified, and patients in the greater reduction group exhibited more favorable cognitive trajectories, particularly with slower progression in CDR‐SB scores; and (3) baseline clinical factors, particularly hypertension, were associated with a diminished biomarker response, suggesting a potential influence of vascular burden on treatment effectiveness. Taken together, these findings provide real‐world evidence supporting the utility of plasma p‐tau217 as an early response biomarker and highlight the potential for biomarker‐guided patient stratification in disease‐modifying therapy.

In the present study, plasma p‐tau217 levels showed a significant and early reduction beginning at 3 months after treatment initiation, with the greatest decline observed between 3 and 6 months, followed by a plateau thereafter. This temporal pattern is broadly consistent with prior studies of anti‐amyloid therapies, in which downstream tau‐related biomarkers exhibit early but time‐dependent changes following amyloid clearance.^6^, ^7^ The p‐tau217 is considered a T1 biomarker that reflects both amyloid and tau pathology.^4^ In the early stages of AD, p‐tau217 levels are known to correlate more strongly with amyloid burden, while still capturing aspects of tau‐related neurodegeneration.^5^, ^17^ Therefore, the early reduction in p‐tau217 observed in our study may primarily reflect the decrease in amyloid‐related pathological processes induced by treatment. In contrast, the subsequent plateauing of p‐tau217 levels after 6 months may be explained by the persistence of underlying tau pathology, which is less directly and more slowly affected by anti‐amyloid therapy. This interpretation is consistent with the concept that amyloid reduction may precede and only partially modulate downstream tau dynamics, resulting in an early decline followed by stabilization of p‐tau217 levels.^6^ These findings further support the utility of plasma p‐tau217 as an early pharmacodynamic marker reflecting upstream amyloid modulation with partial downstream tau‐related effects.

Of note, we identified distinct trajectory patterns of plasma p‐tau217 change, highlighting substantial heterogeneity in biomarker response among patients treated with lecanemab. Notably, patients in the greater reduction group exhibited more favorable cognitive trajectories compared with those in the lesser reduction group, particularly with a slower rate of progression in CDR‐SB scores. These findings suggest that the magnitude of early biomarker reduction may reflect underlying differences in disease biology or treatment responsiveness, and may serve as an indicator of subsequent clinical outcomes.^18^ The observed association between p‐tau217 dynamics and cognitive trajectories underscores the potential clinical relevance of early biomarker response. Although anti‐amyloid therapies primarily target upstream amyloid pathology, our results suggest that downstream biomarker changes, as reflected by p‐tau217, may capture meaningful variation in treatment effect that is not fully explained by baseline clinical characteristics. In this context, p‐tau217 trajectories may provide incremental information beyond conventional measures in identifying patients who are more likely to derive clinical benefit. These findings support the emerging concept of biomarker‐guided stratification in AD.^19^ Early changes in plasma p‐tau217 may help identify responders and non‐responders, enabling more individualized treatment strategies. Such an approach may be particularly valuable in real‐world settings, where patient populations are more heterogeneous than those included in clinical trials. In clinical practice, insufficient early reduction in plasma p‐tau217 may prompt closer reassessment of vascular risk factors, treatment adherence, and concomitant pathologies, along with consideration of additional biomarker or imaging evaluations. In such patients, more intensive non‐pharmacological interventions, including cognitive training and vascular risk optimization, may also be considered. As additional disease‐modifying therapies become available, biomarker‐guided treatment modification strategies may become feasible in patients showing suboptimal biological response.

We further identified that baseline clinical factors, particularly hypertension, were associated with a diminished biomarker response. These findings suggest that vascular burden may play an important role in modulating the biological effects of anti‐amyloid therapy. Hypertension, as a major contributor to cerebrovascular pathology, has been linked to blood–brain barrier dysfunction, impaired perivascular clearance, and reduced glymphatic function, all of which may interfere with the efficient removal of amyloid and downstream modulation of tau‐related processes.^20^, ^21^, ^22^, ^23^ Alternatively, the association between hypertension and attenuated p‐tau217 reduction may partly reflect greater underlying tau pathology in hypertensive patients. Previous studies have shown that elevated blood pressure is associated with higher tau PET burden and more rapid tau accumulation in preclinical AD.^24^, ^25^ Given that plasma p‐tau217 reflects both amyloid and tau‐related pathology, higher baseline tau burden may contribute to the relatively diminished biomarker response observed in patients with hypertension. In addition, younger age was associated with a smaller reduction slope in plasma p‐tau217 levels. Although the underlying mechanisms remain to be fully elucidated, this finding may reflect biological heterogeneity in early‐onset AD, which has been associated with more aggressive tau propagation.^26^, ^27^ It may also help explain prior real‐world observations suggesting that younger patients could exhibit a relatively attenuated clinical response to anti‐amyloid therapy, although this remains somewhat controversial.^3^, ^28^, ^29^ Taken together, these results support the notion that underlying vascular burden and patient‐specific factors may attenuate treatment responsiveness, even in patients who meet clinical criteria for early AD. Of note, these findings highlight the potential need to consider vascular comorbidities and demographic characteristics when evaluating treatment response and may have implications for patient selection and risk stratification in real‐world clinical practice.

The strengths of this study include its prospective real‐world design and the comprehensive evaluation of plasma p‐tau217 dynamics, trajectory patterns, and their association with clinical outcomes in patients with early AD treated with lecanemab. In addition, we applied complementary analytical approaches, including trajectory‐based and slope‐based analyses, to robustly assess biomarker response and its clinical relevance. However, several limitations should be acknowledged. First, this was a single‐center study, which may limit generalizability to other clinical settings. Second, the sample size was modest, particularly for subgroup and biomarker analyses, and the duration of cognitive follow‐up was relatively short, thereby limiting statistical power and precluding conclusions regarding long‐term treatment effects. Third, clustering and slope‐based subgroup analyses were exploratory and may be sensitive to methodological assumptions, including cutoff selection. Fourth, direct measures of tau aggregate pathology, such as tau PET imaging or plasma/cerebrospinal fluid (CSF) microtubule binding region–tau243 (MTBR‐tau243) measurements, were not available in the present cohort. Therefore, the potential contribution of underlying tau burden to differential p‐tau217 response, particularly in relation to hypertension, could not be directly evaluated. Finally, heterogeneity in patient characteristics inherent to real‐world populations may have influenced both biomarker and cognitive outcomes. Despite these limitations, this study provides important real‐world evidence on early biomarker dynamics and their clinical implications in patients receiving lecanemab. Notably, our findings highlight the potential role of plasma p‐tau217 as an early pharmacodynamic marker and support its use in identifying differential treatment responses. Of note, this study adds real‐world data from an Asian cohort, an underrepresented population in pivotal anti‐amyloid trials, underscoring the value of population‐specific evidence for clinical decision‐making.

In conclusion, this prospective real‐world study demonstrates that plasma p‐tau217 levels show an early and sustained reduction following lecanemab treatment and that heterogeneity in biomarker response is associated with differential cognitive trajectories. These findings support the clinical utility of plasma p‐tau217 as a dynamic biomarker of treatment response and may contribute to the development of biomarker‐guided, individualized therapeutic strategies in real‐world practice.

## AUTHOR CONTRIBUTIONS

Concept and design: Sung Hoon Kang. Acquisition of data: Sung Hoon Kang, Yu Jeong Park, Seungyun Lee, Jimin Kang, Seongin Lee, Eun Seong Lee, Hye Na Jung, Inseon Ryoo, Hyundoo Hwang, Kwangho Choi, Jae Seon Eo, Sang‐Il Suh, Kyungmi Oh, and Seong‐Beom Koh. Analysis and interpretation of data: Sung Hoon Kang and Yu Jeong Park. Drafting of the manuscript: Sung Hoon Kang and Yu Jeong Park. Critical revision of the manuscript for important intellectual content: Sung Hoon Kang. Statistical analysis: Sung Hoon Kang, Yu Jeong Park. Supervision: Sung Hoon Kang.

## CONFLICT OF INTEREST STATEMENT

The authors declare no conflicts of interest. Author disclosures are available in the Supporting Information.

## ETHICAL APPROVAL

The institutional review board of Korea University Guro Hospital approved this study. Written informed consent was obtained from all participants prior to study participation, and the data were collected in accordance with the Declaration of Helsinki.

## Supporting information




**Supporting Information**: alz71705‐supp‐0001‐SuppMat.docx

## Data Availability

The datasets generated and analyzed during the current study are available from the corresponding authors upon reasonable request.
