# Supplementary material for: Heterogeneity in plasma p‐tau217 response and its association with cognitive trajectories under lecanemab treatment
Source: Alzheimers Dement. 2026 Jul 31;22(8):e71705. doi: 10.1002/alz.71705 (PMC13425613; doi:10.1002/alz.71705)
Supplement: Supplementary file 1 — Supporting Information: alz71705‐supp‐0001‐SuppMat.docx [file ALZ-22-e71705-s001.docx]

**Supplementary Methods**

**MRI acquisition**

All MRI scans were performed using a 3.0 T MRI scanner (Philips 3.0T Ingenia Elition X; Philips Healthcare, Andover, MA, USA). The baseline MRI protocol included three-dimensional T1-weighted imaging (3D T1WI), three-dimensional fluid-attenuated inversion recovery imaging (3D FLAIR), T2-weighted imaging, diffusion-weighted imaging (DWI), and susceptibility-weighted imaging (SWI) sequences. Follow-up MRI scans were acquired using the same scanner and identical imaging protocol as at baseline.

Baseline MRI was used to assess treatment eligibility and safety, including evaluation of white matter hyperintensities (WMH), the number of cerebral microhemorrhages, the presence of macrohemorrhage, cortical superficial siderosis (cSS), the number of lacunes, and the presence of territorial infarction.

Follow-up MRI scans were evaluated for the occurrence of amyloid-related imaging abnormalities with edema or effusions (ARIA-E) and amyloid-related imaging abnormalities with hemorrhage (ARIA-H). ARIA-E was defined as vasogenic edema or sulcal effusion, and ARIA-H as new or worsening cerebral microhemorrhages, macrohemorrhage, or cortical superficial siderosis compared with baseline MRI. The severity of ARIA-E and ARIA-H was graded as mild, moderate, or severe according to established criteria.^1^ All MRI scans were independently reviewed by two experienced readers (a neuroradiologist and a neurologist), both blinded to all clinical information, and discrepancies in interpretation were resolved by consensus.

**Amyloid PET acquisition and quantification**

Amyloid PET imaging using 18F-florbetaben was performed prior to the initiation of lecanemab treatment to assess eligibility.^1^ For most patients, PET scans were acquired using a Discovery MI PET/computed tomography (CT) scanner (GE Healthcare, Waukesha, WI, USA). A 20-min emission PET scan was performed 90 min after injecting a mean dose of 296 MBq of 18F-florbetaben. Three-dimensional PET images were reconstructed in a 384 × 384 matrix with 0.65 × 0.65 × 2·79 mm voxel size using the ordered-subsets expectation maximization algorithm (iteration = 8 and subset = 34) with time-of-flight and point-spread-function.

All PET scans were independently reviewed by two experienced reviewers (one nuclear medicine physician and one neurologist), and images were dichotomized as amyloid positive or negative based on visual reads, in accordance with established appropriate use criteria.^2,3^ Any discrepancies were resolved by consensus.

All PET images were analyzed with BTXBrain software platform (v1.1.2 Brightonix Imaging Inc., Seoul, Republic of Korea) for Centiloid (CL) quantification.^4,5^ The automated quantified values were provided using PET images alone, bypassing the requirement for CT or MRI. Standardized uptake value ratio (SUVR) was calculated with whole cerebellum as a reference region, and then converted to CL values.

**Supplementary Table 1.** Baseline Clinical and Biomarker Characteristics of Patients Treated with Lecanemab

|  | Lecanemab (N=153) |
| --- | --- |
| Age, years | 72.7 ± 8.3 |
| Female, n (%) | 92 (60.1) |
| Education, years | 11.8 ± 4.7 |
| MCI : mild dementia : SCD, n (%) | 78 (51.0) : 60 (39.2) : 15 (9.8) |
| CDR Global=0.5, n (%) | 117 (76.5) |
| CDR-SB | 3.02 ± 1.95 |
| MMSE | 23.6 ± 3.7 |
| APOE ε4 carrier | 85 (55.6) |
| Hypertension, n (%) | 53 (34.6) |
| Diabetes, n (%) | 28 (18.3) |
| Hyperlipidemia, n (%) | 67 (43.8) |
| CL values | 70.70 ± 35.31 |
| p-tau217 level | 1.19 ± 0.61 |

Values are presented as mean ± standard deviation for continuous variables and number (%) for categorical variables.

Abbreviations: APOE ε4, apolipoprotein E ε4; CDR, Clinical Dementia Rating; CDR-SB, Clinical Dementia Rating–Sum of Boxes; CL, centiloid; MCI, mild cognitive impairment; MMSE, Korean version of the Mini-Mental State Examination, Second Edition; SCD, subjective cognitive decline.

**Supplementary Table 2.** Sensitivity analyses evaluating the incremental prognostic value of longitudinal plasma p-tau217 trajectory groups beyond baseline factors

|  | Baseline factor-based interaction model | Baseline factor +  p-tau217 trajectory model | Likelihood  ratio test |
| --- | --- | --- | --- |
|  | *p*-value | *p*-value | *p*-value |
| MMSE |  |  |  |
| Time × trajectory group | NA | <0.001 |  |
| Time × baseline MMSE | <0.001 | <0.001 |  |
| Time × baseline CDR-SB | 0.010 | 0.005 |  |
| Time × baseline CL | 0.654 | 0.526 |  |
| Time × baseline p-tau217 | 0.053 | 0.110 |  |
| AIC | 1123.8 | 1113.0 |  |
| Model fit comparison |  |  | 0.001 |
| CDR-SB |  |  |  |
| Time × trajectory group | NA | 0.001 |  |
| Time × baseline MMSE | 0.013 | 0.129 |  |
| Time × baseline CDR-SB | 0.312 | 0.383 |  |
| Time × baseline CL | 0.169 | 0.202 |  |
| Time × baseline p-tau217 | 0.746 | 0.937 |  |
| AIC | 548.5 | 534.8 |  |
| Model fit comparison |  |  | <0.001 |

Baseline factor-based interaction models included interactions between time and baseline MMSE, baseline CDR-SB, baseline CL values, and baseline plasma p-tau217 levels after adjustment for age, sex, and APOE ε4 carrier status. Baseline factor+p-tau217 trajectory models additionally included the time-by-p-tau217 trajectory group interaction term. Model fit comparison was performed using likelihood ratio tests between baseline and trajectory models. Lower AIC values indicate improved model fit.

Abbreviations: AIC, Akaike information criterion; CL, centiloid; CDR-SB, Clinical Dementia Rating–Sum of Boxes; MMSE, Korean version of the Mini-Mental State Examination, Second Edition; NA, not applicable.

**
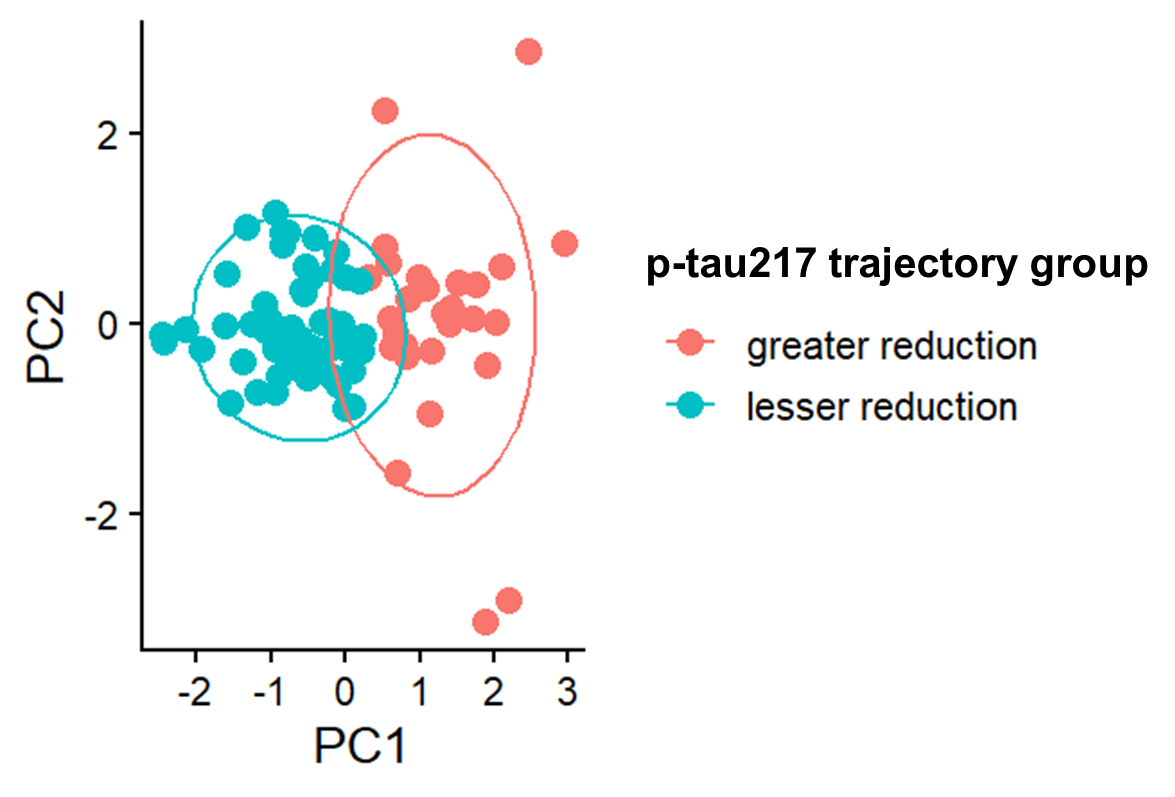
**

**Supplementary Figure 1. Visualization of clustering based on longitudinal changes in plasma p-tau217**

Principal component analysis (PCA) was performed using baseline-normalized changes in plasma p-tau217 levels to visualize separation between trajectory groups. Each point represents an individual participant, colored according to p-tau217 trajectory group (greater reduction vs. lesser reduction). Ellipses indicate the distribution of each group. The two groups show a clear tendency toward separation in the PCA space, supporting the validity of the trajectory-based classification.


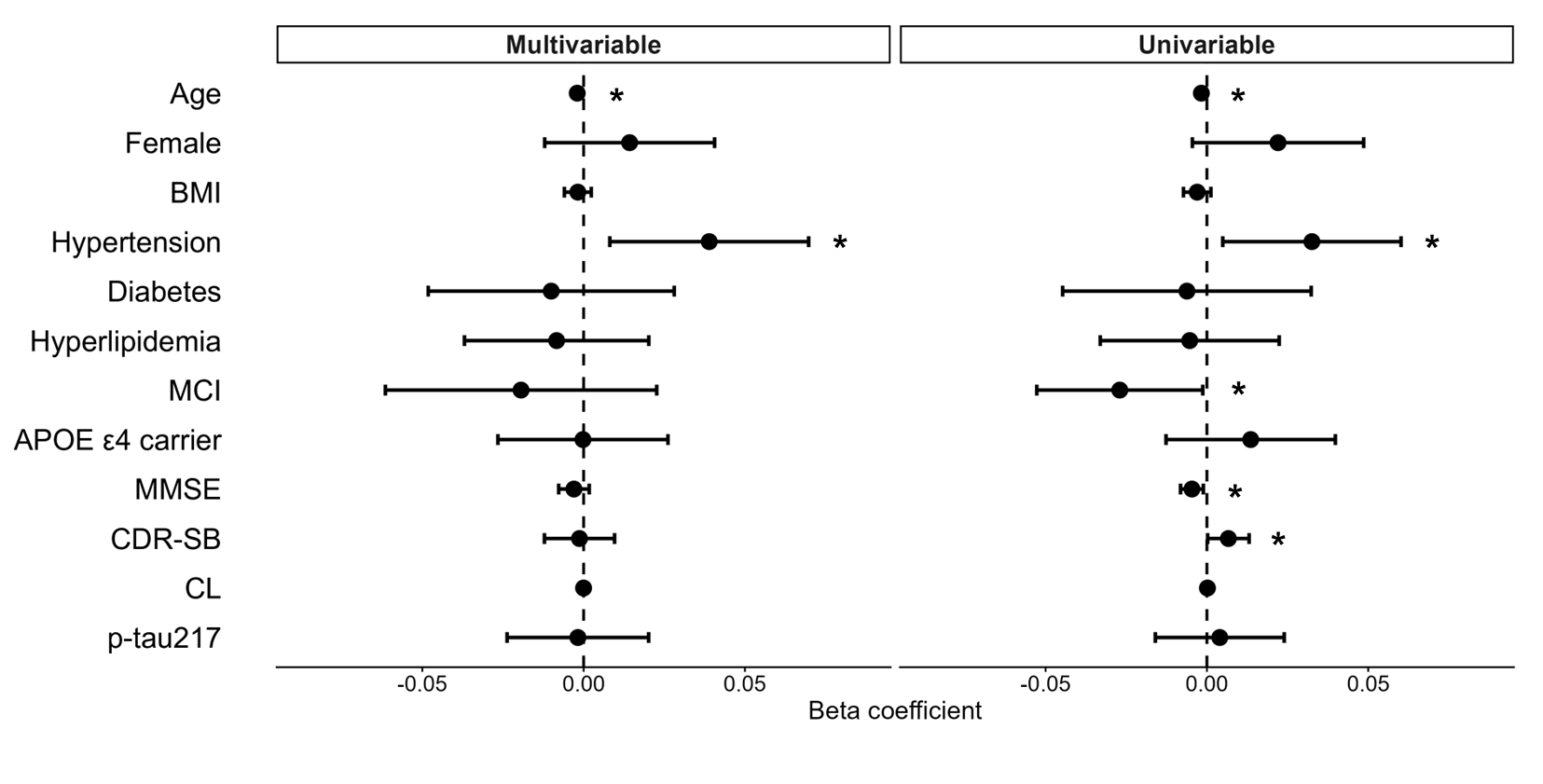
**Supplementary Figure 2. Factors associated with early changes in plasma p-tau217 (0–6 month slope)**

Forest plots show the associations between baseline clinical variables and the 0–6 month slope of plasma p-tau217 levels. *β* coefficients and 95% confidence intervals are presented for multivariable (left panel) and univariable (right panel) linear regression analyses. The dashed vertical line represents the null value (β = 0). A negative *β* indicates a greater reduction (steeper decline) in p-tau217 levels over time, whereas a positive *β* indicates a lesser reduction or relative increase. Asterisks (*) denote statistical significance (*p* < 0.05).

Abbreviations: APOE ε4, apolipoprotein E ε4; BMI, body mass index; CDR-SB, Clinical Dementia Rating–Sum of Boxes; CL, centiloid; MCI, mild cognitive impairment; MMSE, Korean version of the Mini-Mental State Examination, Second Edition.


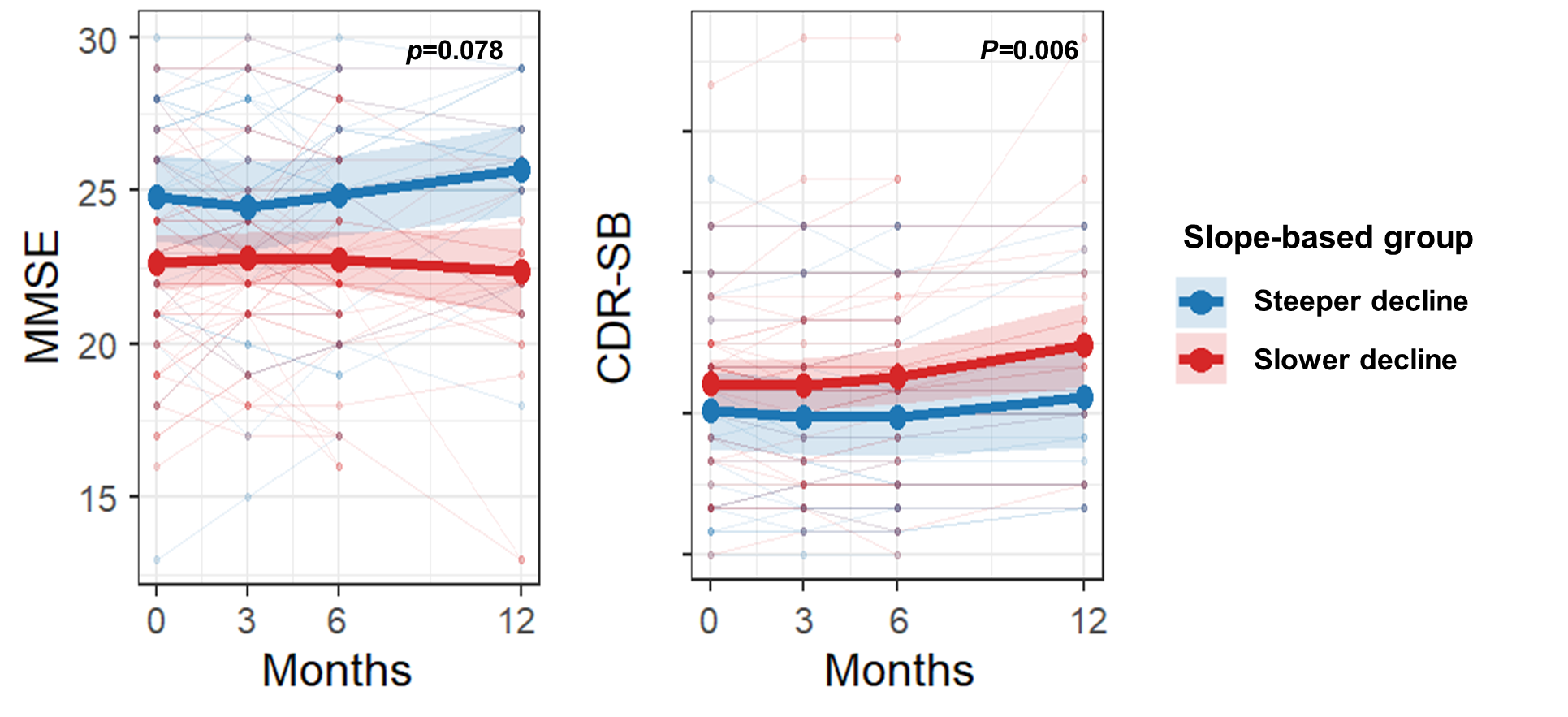


**Supplementary Figure 3. Longitudinal cognitive trajectories according to slope-based p-tau217 groups**

Spaghetti plots illustrate individual longitudinal changes in MMSE (left) and CDR-SB (right) scores (faint lines), with group-level mean trajectories and 95% confidence intervals overlaid (solid lines and shaded areas). Participants were categorized into slope-based groups (steeper decline vs. slower decline) based on the magnitude of 0–6 month changes in plasma p-tau217 levels. Linear mixed-effects models adjusted for age, sex, APOE ε4 genotype, baseline cognitive severity (CDR-SB), baseline plasma p-tau217 levels, and baseline centiloid (CL) values were used to assess differences in longitudinal cognitive changes between groups. *p*-values represent the significance of the interaction between time and slope-based group membership. A significant interaction indicates that the rate of cognitive change differed between groups over time.

Abbreviations: APOE ε4, apolipoprotein E ε4; CDR-SB, Clinical Dementia Rating–Sum of Boxes; CL, centiloid.

**References**

1. Cummings J, Apostolova L, Rabinovici GD, et al. Lecanemab: Appropriate Use Recommendations. *J Prev Alzheimers Dis.* 2023;10:362-377. https://doi.org/10.14283/jpad.2023.30

2. Johnson KA, Minoshima S, Bohnen NI, et al. Appropriate use criteria for amyloid PET: a report of the Amyloid Imaging Task Force, the Society of Nuclear Medicine and Molecular Imaging, and the Alzheimer's Association. *J Nucl Med.* 2013;54:476-490. https://doi.org/10.2967/jnumed.113.120618

3. Kang SH, Kim ME, Jang H, et al. Amyloid Positivity in the Alzheimer/Subcortical-Vascular Spectrum. *Neurology.* 2021;96:e2201-e2211. https://doi.org/10.1212/wnl.0000000000011833

4. Kang SK, Heo M, Chung JY, et al. Clinical Performance Evaluation of an Artificial Intelligence-Powered Amyloid Brain PET Quantification Method. *Nucl Med Mol Imaging.* 2024;58:246-254. https://doi.org/10.1007/s13139-024-00861-6

5. Kang YK, Min JW, Kwon SJ, Ha S. Reliability of Automated Amyloid PET Quantification: Real-World Validation of Commercial Tools Against Centiloid Project Method. *Tomography.* 2025;11 https://doi.org/10.3390/tomography11080086
